# Pedometer determined physical activity tracks in African American adults: The Jackson Heart Study

**DOI:** 10.1186/1479-5868-9-44

**Published:** 2012-04-18

**Authors:** Robert L Newton, Hongmei Han M, Patricia M Dubbert, William D Johnson, DeMarc A Hickson, Barbara Ainsworth, Teresa Carithers, Herman Taylor, Sharon Wyatt, Catrine Tudor-Locke

**Affiliations:** 1 Pennington Biomedical Research Center, Baton Rouge, LA, USA; 2 South Central VA Mental Illness Research Education & Clinical Center, Little Rock, AR, USA; 3 Little Rock Geriatric Research Education & Clinical Center, Little Rock, AR, USA; 4 Psychiatric Research Institute, University of Arkansas for Medical Sciences, Little Rock, AR, USA; 5 Jackson State University, Jackson, MS, USA; 6 University of Mississippi Medical Center, Jackson, MS, USA; 7 Arizona State University, Phoenix, AZ, USA; 8 University of Mississippi, University, Mississippi, MS, USA

**Keywords:** Physical activity assessment, African Americans, Sedentary, Validity

## Abstract

**Background:**

This study investigated the number of pedometer assessment occasions required to establish habitual physical activity in African American adults.

**Methods:**

African American adults (mean age 59.9 ± 0.60 years; 59 % female) enrolled in the Diet and Physical Activity Substudy of the Jackson Heart Study wore Yamax pedometers during 3-day monitoring periods, assessed on two to three distinct occasions, each separated by approximately one month. The stability of pedometer measured PA was described as differences in mean steps/day across time, as intraclass correlation coefficients (ICC) by sex, age, and body mass index (BMI) category, and as percent of participants changing steps/day quartiles across time.

**Results:**

Valid data were obtained for 270 participants on either two or three different assessment occasions. Mean steps/day were not significantly different across assessment occasions (p values > 0.456). The overall ICCs for steps/day assessed on either two or three occasions were 0.57 and 0.76, respectively. In addition, 85 % (two assessment occasions) and 76 % (three assessment occasions) of all participants remained in the same steps/day quartile or changed one quartile over time.

**Conclusion:**

The current study shows that an overall mean steps/day estimate based on a 3-day monitoring period did not differ significantly over 4 – 6 months. The findings were robust to differences in sex, age, and BMI categories. A single 3-day monitoring period is sufficient to capture habitual physical activity in African American adults.

## Background

National data indicate that most Americans do not engage in the recommended amount of regular physical activity (PA) [1,2]. Low levels of PA have been shown to be associated with chronic disease, such as diabetes, cardiovascular disease, and certain cancers [3,4]. Most of the conclusions in these studies were based on self-reported PA. However, with the advent of personalized motion detecting devices (e.g. pedometers, accelerometers), research has shifted towards re-examining PA levels using objective measures. Despite the fact that research consistently shows that African American adults engage in low levels of self-reported exercise [5,6] and have low levels of physical fitness [7,8], very little objective data exist on PA behaviors in African American adults [9,10]. This lack of objectively measured habitual PA limits our ability to confirm findings [11-13] or illuminate the true relationship between PA and chronic disease in African American adults [14]. In this population, health outcomes such as diabetes, hypertension, cardiovascular diseases (e.g. coronary heart disease, strokes) and certain cancers are of paramount importance because they are considered principal sources of health disparities [15-19].

Several studies have objectively assessed PA in African American adults via pedometry [20-25]. The average daily step count in these studies has ranged from 4,355 [23] to 7,654 steps/day [24]. According to Tudor-Locke and Bassett [26], African American adults would be categorized as sedentary on average (i.e., taking < 5,000 steps/day) in two [23,20] of the five referenced studies, and in none of the five would they be classified as active (i.e., taking ≥ 10,000 steps/day). Data in all but one [25] of these studies in African American adults were derived from a single assessment occasion (i.e., multiple days of monitoring over a single brief interval of time) spanning three days [21] to two weeks [23]. However, there are no data in the current literature to indicate that a single assessment occasion is sufficient to provide a reliable estimate of habitual PA in African American adults.

Assessment of PA by pedometry is reliable and accurate [27,28]. However, Kang et al. [29] have suggested that numerous (and randomly selected) assessment occasions are necessary to establish a precise estimate of habitual PA, although these conclusions were based on modeling the number and patterns of single day values against a year-long average criterion. Specifically, these researchers concluded that a minimum of 30 consecutive days or 14 randomly selected days throughout the year were necessary to ensure valid (i.e., relative to the year-long average) and stable estimates of habitual PA [29]. However, longer and more complex monitoring periods are burdensome to both study participants and researchers, and therefore, shorter assessment periods are desirable in pragmatic research. Tracking of PA across multiple, short, assessment occasions may be a better approach to evaluating habitual PA compared to a single, extended assessment period. In two clinical studies [30,31], a 3-day monitoring period (any combination of days in a week) provided stable estimates (Intraclass Correlation Coefficient (ICC) ≥ .80) of PA obtained over the course of the full week. However, the longer-term stability of a 3-day monitoring period is not known.

To date, no study has attempted to characterize the stability of habitual PA in an African American adult population. The Jackson Heart Study (JHS) [32], an epidemiological study of cardiovascular disease in African American adults, provides an ideal opportunity to accomplish this goal. The JHS enrolled 5,301 adults, of which a subset of participants wore pedometers for a 3-day monitoring period (consecutive days) on two to three distinct occasions, each separated by approximately one month [33]. The current analyses used data from the JHS to assess the stability of pedometer-determined estimates of PA. Conclusions will inform the appropriateness of using a single assessment occasion in future studies that examine relationships between PA and health outcomes in this population.

## Methods

### Jackson Heart Study

The JHS is a longitudinal population-based study designed to study novel causes of cardiovascular and other chronic diseases in African American adults [32]. Between 2000 and 2004, 5,301 African American men and women between the ages of 21 and 95 years were recruited from the Jackson, MS metropolitan statistical area, including Hinds, Madison, and Rankin counties. Recruitment details have been described elsewhere [34]. Three academic institutions have collaborated on the project, including Jackson State University, the University of Mississippi Medical Center, and Tougaloo College. The research was approved by the Institutional Review Boards of the University of Mississippi Medical Center, the Jackson State University, and Tougaloo College. All participants provided written informed consent.

### Participants

The participants for the current analysis represent those who agreed to wear pedometers as part of the Diet and Physical Activity Substudy (DPASS) of the JHS [35]. The DPASS was designed to validate the dietary and PA instruments that were utilized in the JHS.

### Anthropometrics

Body mass index (BMI). Height was measured without shoes and recorded to the nearest centimeter. Participants stood with their feet together and head held in the Frankfurt plane. Weight was measured on a balance scale, in light clothing, without shoes, and recorded to the nearest 0.5 kilogram. BMI was calculated as weight in kilograms divided by height in square meters.

### Pedometer assessment

As mentioned above, pedometer-determined PA was assessed as part of the DPASS. The DPASS involved five clinic visits in which participants completed both dietary and PA recalls (Table 1). A week before their second scheduled clinic visit participants were mailed a Yamax SW-200 pedometer (Yamax Corp., Tokyo, Japan) and instructed to wear it at their waist, attached to the waistband of their clothing or belt for a 3-day (consecutive) monitoring period. They were also mailed a step log to record daily steps at the end of the day. Participants were asked to reset the pedometer at the beginning of each day and to remove it only at night for sleeping or for water activities, such as bathing or swimming. Participants were also instructed to record times when the device was not worn. Participants returned the log and the pedometer at the subsequently scheduled clinic visit. This procedure was repeated for a maximum of three separate pedometer assessment occasions. Each of the DPASS clinic visits was separated by approximately one month.

**Table 1:** Schedule of DPASS clinic visits

**1^st ^DPASS clinic visit**	**~ 1 month period**	**2^nd ^ DPASS clinic visit**	**~ 1 month period**	**3^rd^ DPASS clinic visit**	**~ 1 month period**	**4^th ^ DPASS clinic visit**	**~ 1 month period**	**5^th ^ DPASS clinic visit**
Dietary/physical activity questionnaires	Pedometer worn for 3 days prior to 2^nd ^clinic visit	Pedometer data collected	Pedometer worn for 3 days prior to 3^rd ^clinic visit	Pedometer data collected	Pedometer worn for 3 days prior to 4^th ^clinic visit	Pedometer data collected	Pedometer worn for 3 days prior to 5^th ^clinic visit†	Pedometer data collected‡

†Pedometers were only worn during this one month time frame if pedometer data had not been collected during the previous three clinic visits.

‡Pedometer data were only collected during this visit if pedometers were worn the three days prior to this clinic visit.

DPASS: Diet and physical activity sub-study.

### Statistical Analysis

This analysis focused on tracking of pedometer-determined PA, and therefore, participants must have completed at least two valid assessment occasions. A valid assessment occasion was defined as having all three required days of pedometer data. There were 481 participants with pedometer data. Data from any participant who had < 500 steps per day were removed from the analyses. This resulted in 91 participants being excluded due to a lack of any valid assessment occasions. Another 115 participants were excluded because they only had a single valid assessment occasion. An additional 5 participants were excluded because of missing sex, age, and/or BMI data. After these exclusions, a total of 270 participants had usable data for the purposes of the current analyses. Of the total analyzable sample, 137 participants had two valid assessment occasions and 133 had three valid assessment occasions. Steps/day was averaged across the 3-day monitoring period for each assessment occasion. It should be noted that 46 % of the participants with two valid assessment occasions, and 44 % of the participants with three valid assessment occasions wore the pedometer on at least one weekend day across the assessment occasions.

Stability in steps/day was assessed in several ways. First, the average steps/day was compared across assessment occasions using a paired *t*-test for those with two valid assessment occasions, and repeated measures ANOVA was used for those with three valid assessment occasions. Second, separate ICCs were calculated by number of valid assessment occasions, as well as by sex, age (age ≤ 60 years vs age > 60 years), and BMI (BMI < 30 kg/m^2^ vs BMI ≥ 30 kg/m^2^) categories. The median value was used to dichotomize age groups, and BMI was dichotomized at the value used to define obesity (BMI = 30). ICCs were used to quantify the tendency for a participant’s steps/day to be the same when measured on different occasions and thus to assess PA stability across assessment occasions. The variability in steps/day can be expressed as the sum, *v*_*b*_ + *v*_*w*_, where *v*_*b*_ is a component of variability attributable to differences among occasions, that is differences from occasion to occasion, and *v*_*w*_ is a component attributable to variability among participants within occasions. The ICC can be expressed as the proportion *v*_*b*_*/(v*_*b*_ + *v*_*w*_) with possible values ranging from 0 to 1. The minimum ICC value (*complete instability*) is realized when all the variability is attributable to within participant variance, i.e., *v*_*b*_ *=* 0, *v*_*w*_ *>* 0; and the maximum value *(complete stability*) is realized when all the variability is attributable to among occasion variance, i.e., *v*_*b*_ *>* 0, *v*_*w*_ = 0. Malina’s [36] criteria for strength of the ICC were used as a guide in interpreting observed ICC values: ICC values < 0.30 indicated weak stability; values 0.30 – 0.59 indicated moderate stability; values ≥ 0.60 indicated moderately high stability. Relatedly, the changes in steps/day in absolute values were calculated and expressed in percentage for each individual. The medians were reported for this variable due to skewness of the data. For two valid assessment occasions, the percentage was calculated as the absolute difference between the mean steps/day for the first and second valid assessment occasions and dividing by the mean steps/day for the first assessment occasion. For three valid assessment occasions, a similar calculation was performed for each of three changes (i.e. mean steps/day for valid assessment occasion 2 to 1, 3 to 2, and 3 to 1) and the average was utilized. Third, a descriptive approach was used. The participants were divided into quartiles based on average steps/day. Participants who remained in the same quartile across the valid assessment occasions were considered ‘stable’, those who switched one quartile were considered ‘moderately stable’, those who switched two quartiles were considered ‘moderately unstable’, and those who switched three quartiles were considered ‘unstable.’

The following power analysis was based on a one sample *t*-test (time 1 vs follow up) design. Using a standard deviation of 3000 and an alpha of .05 one should have 85 % power to detect a change of 550 steps, if 390 subjects at baseline, assuming 30 % incomplete data. Similarly, one should have 83 % power to detect a change of 1100 steps for analyses by gender and by BMI group. All analyses were performed in SAS 9.2 (SAS institute, Cary, NC).

## Results

Descriptive characteristics for the participants are presented in Table 2. There were no differences in age and BMI between participants in the various assessment occasion categories, but steps/day was greater in those with three valid assessment occasions compared to those with one or two. The overall mean change in steps/day for participants with two valid assessment occasions was −26.5 (95 % CI: –560.3, 507.3) and was 203.0 (95 % CI: –276.4, 682.4) for those with three valid assessment occasions. Table 3 shows that steps/day means did not differ significantly between valid assessment occasions for the overall sample or within categories of sex, age, or BMI, for either two (all p values > 0.234) or three valid assessment occasions (all p values > 0.097). The overall ICCs for two and three valid assessment occasions were 0.57 and 0.76, respectively, indicating moderate and a moderately high stability of steps/day across time periods according to Malina’s [36] criteria. The ICCs stratified within categories of sex, age, and BMI status ranged from 0.43 to 0.60 for two valid assessment occasions, and 0.65 to 0.82 for three valid assessment occasions. 

**Table 2:** JHS DPASS (n = 270) participant characteristics by number of valid assessment occasions

**Variable**	**Number of valid assessment occasions**									
	**One**			**Two**			**Three**			
**Men**	37.4 %			38.7 %			44.4 %			
	**M ± SE**	**Min**	**Max**	**M ± SE**	**Min**	**Max**	**M ± SE**	**Min**	**Max**	**p**
**Age (years)**	60.1 ± 0.88	39.0	80.0	60.5 ± 0.82	37.0	77.0	59.3 ± 0.88	37.0	81.0	0.57
**BMI (kg/m**^**2**^**)**	31.8 ± 0.65	20.2	65.1	31.0 ± 0.65	18.3	64.3	29.9 ± 0.45	16.0	45.1	0.087
**Steps/day**	4761.1± 297.1^a^	652.7	15273.7	5327.8± 259.4^a^	827.7	20304.8	6671.3± 322.2^b^	1424.7	22478.7	<0.001

Note: A valid assessment occasion was defined as having all three days of pedometer data. BMI = Body mass index.

Values with dissimilar superscripts are significantly different from one another.

**Table 3:** **Mean steps/day and percent change in absolute values expressed as median for participants with two and three valid assessment occasions**^**a**^

	**N**	**M ± SE**	**M ± SE**	**M ± SE**	**Change (95 % CI)**	**Median % change in absolute steps (LQ,UQ)**	**p-value**	**ICC**
**Two valid assessment occasions**	137	5341 ± 282	5315 ± 303	N/A	−26.5 (−560.3, 507.3)	35.4 (20.9, 63.4)	0.922	0.57
**Sex**								
Male	53	6141 ± 505	5818 ± 479	N/A	−322.6 (−1283.7, 638.5)	35.4 (20.3, 60.0)	0.504	0.53
Female	84	4837 ± 322	4997 ± 389	N/A	160.3 (−477.9, 798.6)	34.9 (24.4, 66.8)	0.619	0.60
**Age**								
≤ 60	60	5995 ± 470	6433 ± 499	N/A	437.4 (−467.5, 1342.3)	35.9 (15.4, 64.3)	0.337	0.56
> 60	77	4831 ± 334	4443 ± 344	N/A	−387.9 (−1031.4, 255.5)	34.5 (23.6, 62.6)	0.234	0.54
**BMI**								
< 30	73	5571 ± 399	5513 ± 465	N/A	−57.8 (−766.2, 650.6)	30.2 (17.8, 63.0)	0.871	0.67
≥ 30	64	5079 ± 397	5089 ± 375	N/A	−9.2 (−819.7, 838.2)	44.5 (26.4, 63.8)	0.982	0.43
**Three valid** **assessment** **occasions**	133	6636 ± 351	6539 ± 351	6839 ± 351	203.0 (−276.4, 682.4)	29.9 (20.6, 44.6)	0.456	0.76
**Sex**								
Male	59	7638 ± 604	7384 ± 604	7622 ± 604	−15.2 (−736.3, 705.8)	31.3 (20.4, 41.5)	0.737	0.82
Female	74	5837 ± 394	5866 ± 394	6214 ± 394	377.1 (−273.0, 1027.1)	28.5 (20.7, 48.0)	0.445	0.65
**Age**								
≤ 60	69	7604 ± 540	7442 ± 540	8240 ± 540	635.8 (−130.1, 1401.7)	26.0 (17.9, 48.8)	0.097	0.76
> 60	64	5591 ± 393	5566 ± 393	5328 ± 393	−263.6 (−817.4, 290.3)	31.6 (23.3, 42.6)	0.583	0.74
**BMI**								
< 30	69	6834 ± 495	6812 ± 495	7247 ± 495	413.1 (−257.3, 1083.5)	29.6 (20.2, 43.0)	0.361	0.77
≥ 30	64	6422 ± 450	6238 ± 450	6399 ± 450	−23.4 (−719.1, 672.2)	31.0 (21.2, 44.6)	0.851	0.76

^a^A valid assessment occasion was defined as having all three days of pedometer data.

Note: Paired t-tests were performed for participants who had two valid assessment occasions. Repeated measures ANOVA were performed for participants with three valid assessment occasions. JHS: Jackson Heart Study. ICC: Intraclass correlation coefficient. BMI = Body mass index.

The median of the overall percent change in absolute values for those with two valid assessment occasions was 35.4 % (Q1: 20.9 %; Q4: 63.4 %) and ranged from 30.2 % to 44.5 % for participants differing in sex, age, and BMI status (Table 2). The median of the overall percent change in absolute values was 29.9 % (Q1: 20.6 %; Q4: 44.6 %) for participants with three valid assessment occasions, and ranged from 26.0 % to 31.6 % for participants differing in sex, age, and BMI status. All of these values were significantly different from 0 (p values < .001).

**Figure 1:**
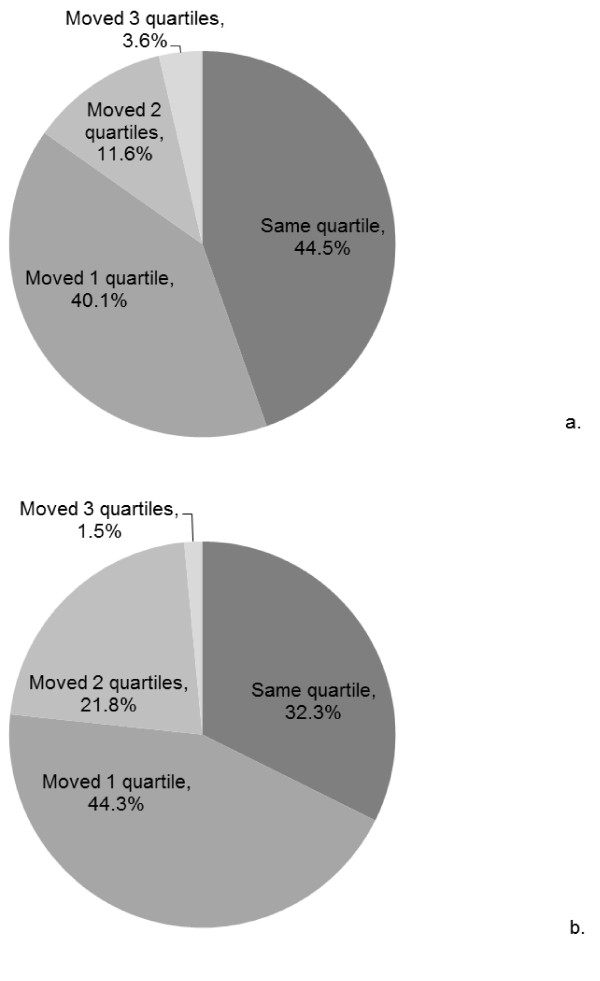
** Stability/instability in steps/day for JHS participants with a) two or b) three valid assessment occasions .**Legend: Participants who remained in the same steps/day quartile across the valid assessment occasions were considered ‘stable’, those who changed one quartile were considered ‘moderately stable’, those who changed two quartiles were considered ‘moderately unstable’, and those who changed three quartiles were considered ‘unstable.’.

Figure 1 illustrates the quartile analysis. Approximately 85 % and 76 % of the participants with two and three valid assessment occasions, respectively, were at least “moderately stable” (remained in the same steps/day quartile or changed one quartile across time) in their activity. The proportion of participants who became more active was equal to the proportion who became less active for both two (p = .636) and three (p = .710) valid assessment occasions.

## Discussion

The analyses of the JHS DPASS data show that a single assessment occasion comprising a 3-day monitoring period of pedometer-determined steps/day provides estimates of PA that are reasonably stable across 4 – 6 months. This was illustrated by the moderate to moderately high ICCs, the fact that between 76 % and 85 % of participants were ‘moderately stable’ or ‘stable’ in their behavior, and that there were no significant differences in mean steps/day across assessment periods. These findings are consistent with those reported in a study of pedometer tracking of steps/day in Australian adults, which utilized a 7-day monitoring period and assessed stability in mean steps/day over one year [37]. In addition, the current study revealed that mean steps/day was reasonably stable within different sex, age, and BMI categories. Although confirmation is needed with larger and more diverse populations, our results suggest that, in the absence of interventions to increase PA, a single shorter (i.e., three days) and less burdensome assessment period can be confidently utilized to provide a reasonably stable estimate of habitual PA over a period of several months.

Our results extend previous reports demonstrating that a 3-day monitoring period accurately reflected activity over a week [30,31] by showing that it provides a reasonably stable estimate of habitual PA over a period of 4 – 6 months. However, there were some differences between those who completed two versus three valid assessment occasions. First, steps/day was lower in participants with only two valid assessment occasions. It has been shown that individuals who are inactive tend to wear activity devices for a fewer number of days and for fewer hours per day compared to those who are active [38]. Second, the ICCs were generally lower in participants with two (overall ICC = 0.57) compared to three (overall ICC = 0.76) valid assessment occasions. Thus, between 57 % and 76 % of the total variability in steps/day is attributed to differences among occasions, with the remainder (about 25 % – 40 %) being attributed to natural shifts in PA patterns within the participants. This is consistent with the fact that the individual variability was within this range (overall percent change in absolute values ranged from 29.9 % to 35.4 %). The lower ICC values indicate that PA may not track as well in those completing two versus three valid assessment occasions. However, in those completing two valid assessment occasions, there were no significant differences in mean steps/day across time and a high proportion (>75 %) were at least ‘moderately stable’. These results suggest that mean steps/day provide an accurate depiction of participant’s personal habitual PA behaviors.

As noted above, previous studies of pedometer-determined steps/day have demonstrated that African Americans take an average of between 4,700 – 7,600 steps/day [20-25], whereas the current study provides slightly higher estimates (5,300 – 6,800 steps/day). Together, these estimates reveal that the average steps/day for African Americans are indicative of either a sedentary (< 5,000 steps/day) or low active lifestyle (5,000 – 7,499 steps/day) [39]. In fact, only 31.1 % of the participants in the current study averaged ≥ 7,000 steps/day, a threshold that has recently been shown to be indicative of accumulating 150 minutes/week of moderate to vigorous physical activity (MVPA) [40]. These JHS data are also consistent with other objective PA data [9,10] (which show prevalence of regular MVPA ranging from 1 % – 38 %) and self-reported PA data [5,6] (which show prevalence of regular MVPA ranging from 24 % – 40 %). The findings indicate that a limited number of African Americans are engaging in the recommended amounts of regular MVPA, and these inadequate levels of PA are placing them at increased risk for developing chronic disease. These multiple sources of PA data should serve as an impetus for developing theoretically sound interventions to promote regular PA in this population.

There are some limitations to the current study which should be taken into account when interpreting the findings. Only 270 of 480 (56 %) participants were included in the final analyses, and only 28 % (133 of 480) of the total DPASS sample provided data at all required DPASS assessment visits. Therefore, only a third of the participants fully complied with the study requirements. The compliant participants were more active and their activity was more stable than the rest of the participants, and therefore, the number of steps/day data may not be generalizable to the entire JHS sample. However, the data show that steps/day are reasonably stable across time regardless of whether participants had two or three assessment occasions. Furthermore, the mean steps/day for participants who completed one and two valid assessments were not significantly different from one another, thus the stability is likely similar between them. Another limitation is that although the use of pedometry provided an objective measure of activity, pedometers are not designed to detect intensity nor type of activity. In addition, the thresholds distinguishing stability vs. instability used herein were based on dividing the participants into steps/day quartiles. Ideally, the threshold would have been set at the level of PA consistent with the current recommendations of 150 minutes per week (>7,000 steps/day) [40], however, too few individuals met this criteria (31.1 %).

## Conclusions

We used pedometers to track PA in African Americans enrolled in the JHS DPASS substudy. The findings are important because they suggest that a single 3-day monitoring period provides sufficient data for reliable estimation of PA over the course of 4 – 6 months in African Americans. In other words, researchers can choose any 3-day assessment occasion and can be confident that it represents habitual PA for this sample. These estimates appear to be robust to differences in sex, age, and BMI. The findings from the current study suggest that the JHS steps/day data are reasonably stable across time and accurately reflect habitual PA in this population. This is significant because it provides the basis for future studies to confidently assess the relationship between objectively measured habitual PA and health outcomes in the JHS, one of the most comprehensive epidemiological studies of cardiovascular disease in African American adults to date.

## Abbreviations

ICC: Intraclass correlation; BMI: Body Mass Index; PA: physical activity; JHS: Jackson Heart Study; DPASS: Diet and Physical Activity Substudy; SE: Standard error; A: Active; I: Inactive; MVPA: moderate to vigorous physical activity.

## Competing interests

The authors declare that they have no competing interests.

## Authors’ contributions

RLNJr participated in the design of the study, interpreted the data, and drafted the manuscript. HH, WJ conducted the statistical analyses. DAH was responsible for data acquisition. PAD conceived the design of the study, acquired data, interpreted the data, and drafted the manuscript. TC, SW, HT, revised the manuscript critically for important intellectual content. CTL helped to conceive and design the study, interpret the data, and draft the manuscript. All authors read and approved the final manuscript.

## References

[B1] Carlson SA Fulton JE Schoenborn CA Loustalot F Trend and prevalence estimates based on the 2008 Physical Activity Guidelines for Americans Am J Prev Med 2010 39 305 313 10.1016/j.amepre.2010.06.006 20837280

[B2] Carlson SA Densmore D Fulton JE Yore MM Kohl HW III Differences in physical activity prevalence and trends from 3 U.S. surveillance systems: NHIS, NHANES, and BRFSS J Phys Act Health 2009 6 1 18 27 10.1123/jpah.6.s1.s18 19998846

[B3] Haskell WL Lee I-M Pate RR Powell KE Blair SN Franklin BA Macera CA Heath GW Thompson PD Physical Activity and Public Health: Updated Recommendation for Adults from the American College of Sports Medicine and the American Heart Association Circulation 2007 39 1423 1434 10.1249/mss.0b013e3180616b27 17762377

[B4] Campbell KL McTiernan A Exercise and biomarkers for cancer prevention studies J Nutr 2007 137 Suppl 1 161 169 10.1093/jn/137.1.161S 17182820

[B5] Macera CA Ham SA Yore MM Jones DA Ainsworth BE Kimsey CD Kohl HW III Prevalence of physical activity in the United States: Behavioral Risk Factor Surveillance System, 2001 Prev Chronic Dis 2005 2 A17 15888228 PMC1327711

[B6] Whitt-Glover MC Taylor WC Heath GW Macera CA Self-reported physical activity among blacks: estimates from national surveys Am J Prev Med 2007 33 412 417 10.1016/j.amepre.2007.07.024 17950407

[B7] Carnethon MR Gulati M Greenland P Prevalence and cardiovascular disease correlates of low cardiorespiratory fitness in adolescents and adults JAMA 2005 294 2981 2988 10.1001/jama.294.23.2981 16414945

[B8] Sidney S Haskell WL Crow R Sternfeld B Oberman A Armstrong MA Cutter GR Jacobs DR Savage PJ Van HL Symptom-limited graded treadmill exercise testing in young adults in the CARDIA study Med Sci Sports Exerc 1992 24 177 183 1549006

[B9] Troiano RP Berrigan D Dodd KW Masse LC Tilert T McDowell M Physical activity in the United States measured by accelerometer Med Sci Sports Exerc 2008 40 181 188 18091006 10.1249/mss.0b013e31815a51b3

[B10] Wolin KY Heil DP Askew S Matthews CE Bennett GG Validation of the International Physical Activity Questionnaire-Short among Blacks J Phys Act Health 2008 5 746 760 18820348 10.1123/jpah.5.5.746 PMC2744347

[B11] Rosenberg L Boggs D Wise LA Palmer JR Roltsch MH Makambi KH ms-Campbell LL: A follow-up study of physical activity and incidence of colorectal polyps in African-American women Cancer Epidemiol Biomarkers Prev 2006 15 1438 1442 10.1158/1055-9965.EPI-06-0079 16896029

[B12] Gillum RF Mussolino ME Madans JH Diabetes mellitus, coronary heart disease incidence, and death from all causes in African American and European American women: The NHANES I epidemiologic follow-up study J Clin Epidemiol 2000 53 511 518 10.1016/S0895-4356(99)00208-5 10812324

[B13] Harris MI Flegal KM Cowie CC Eberhardt MS Goldstein DE Little RR Wiedmeyer HM Byrd-Holt DD Prevalence of diabetes, impaired fasting glucose, and impaired glucose tolerance in U.S. adults The Third National Health and Nutrition Examination Survey, 1988–1994. Diabetes Care 1998 21 518 524 10.2337/diacare.21.4.518 9571335

[B14] Dubbert PM Robinson JC Sung JH Ainsworth BE Wyatt SB Carithers T Newton R Jr Rhudy JL Barbour K Sternfeld B Taylor H Jr Physical activity and obesity in African Americans: the Jackson Heart Study Ethn Dis 2010 20 383 389 21305826 PMC5074338

[B15] Bravata DM Wells CK Gulanski B Kernan WN Brass LM Long J Concato J Racial disparities in stroke risk factors: the impact of socioeconomic status Stroke 2005 36 1507 1511 10.1161/01.STR.0000170991.63594.b6 15961710

[B16] Cowie CC Rust KF Ford ES Eberhardt MS Byrd-Holt DD Li C Williams DE Gregg EW Bainbridge KE Saydah SH Geiss LS Full accounting of diabetes and pre-diabetes in the U.S. population in 1988–1994 and 2005–2006 Diabetes Care 2009 32 287 294 19017771 10.2337/dc08-1296 PMC2628695

[B17] Lloyd-Jones D Adams R Carnethon M De SG Ferguson TB Flegal K Ford E Furie K Go A Greenlund K Haase N Hailpern S Ho M Howard V Kissela B Kittner S Lackland D Lisabeth L Marelli A McDermott M Meigs J Mozaffarian D Nichol G O'Donnell C Roger V Rosamond W Sacco R Sorlie P Stafford R Steinberger J Thom T Wasserthiel-Smoller S Wong N Wylie-Rosett J Hong Y Heart disease and stroke statistics–2009 update: a report from the American Heart Association Statistics Committee and Stroke Statistics Subcommittee Circulation 2009 119 e21 181 19075105 10.1161/CIRCULATIONAHA.108.191261

[B18] Qureshi AI Suri MF Kirmani JF Divani AA Prevalence and trends of prehypertension and hypertension in United States: National Health and Nutrition Examination Surveys 1976 to 2000 Med Sci Monit 2005 11 403 409 16127357

[B19] Delancey JO Thun MJ Jemal A Ward EM Recent trends in Black-White disparities in cancer mortality Cancer Epidemiol Biomarkers Prev 2008 17 2908 2912 10.1158/1055-9965.EPI-08-0131 18990730

[B20] Bennett GG Wolin KY Viswanath K Askew S Puleo E Emmons KM Television viewing and pedometer-determined physical activity among multiethnic residents of low-income housing Am J Public Health 2006 96 1681 1685 10.2105/AJPH.2005.080580 16873736 PMC1551955

[B21] Crane PB Wallace DC Cardiovascular risks and physical activity in middle-aged and elderly African American women J Cardiovasc Nurs 2007 22 297 303 17589282 10.1097/01.JCN.0000278960.82877.91

[B22] Hornbuckle LM Bassett DR Jr Thompson DL Pedometer-determined walking and body composition variables in African-American women Med Sci Sports Exerc 2005 37 1069 1074 15947735

[B23] Panton LB Kushnick MR Kingsley JD Moffatt RJ Haymes EM Toole T Pedometer measurement of physical activity and chronic disease risk factors of obese lower socioeconomic status African American women J Phys Act Health 2007 4 447 458 18209235

[B24] Tudor-Locke C Ainsworth BE Whitt MC Thompson RW Addy CL Jones DA The relationship between pedometer-determined ambulatory activity and body composition variables Int J Obes Relat Metab Disord 2001 25 1571 1578 10.1038/sj.ijo.0801783 11753573

[B25] Whitt MC DuBose KD Ainsworth BE Tudor-Locke C Walking patterns in a sample of African American, Native American, and Caucasian women: the cross-cultural activity participation study Health Educ Behav 2004 31 Suppl 4 45 56 10.1177/1090198104266034 15296691

[B26] Tudor-Locke C Bassett DR Jr How many steps/day are enough? Preliminary pedometer indices for public health Sports Med 2004 34 1 8 10.2165/00007256-200434010-00001 14715035

[B27] Crouter SE Schneider PL Bassett DR Jr Spring-levered versus piezo-electric pedometer accuracy in overweight and obese adults Med Sci Sports Exerc 2005 37 1673 1679 10.1249/01.mss.0000181677.36658.a8 16260966

[B28] Schneider PL Crouter SE Bassett DR Pedometer measures of free-living physical activity: comparison of 13 models Med Sci Sports Exerc 2004 36 331 335 10.1249/01.MSS.0000113486.60548.E9 14767259

[B29] Kang M Bassett DR Barreira TV Tudor-Locke C Ainsworth B Reis JP Strath S Swartz A How many days are enough? A study of 365 days of pedometer monitoring Res Q Exerc Sport 2009 80 445 453 10.5641/027013609X13088500159129 19791630

[B30] Tudor-Locke C Burkett L Reis JP Ainsworth BE Macera CA Wilson DK How many days of pedometer monitoring predict weekly physical activity in adults? Prev Med 2005 40 293 298 10.1016/j.ypmed.2004.06.003 15533542

[B31] Felton GM Tudor-Locke C Burkett L Reliability of pedometer-determined free-living physical activity data in college women Res Q Exerc Sport 2006 77 304 308 10.5641/027013606X13080769704884 17020074

[B32] Taylor HA Jr Wilson JG Jones DW Sarpong DF Srinivasan A Garrison RJ Nelson C Wyatt SB Toward resolution of cardiovascular health disparities in African Americans: design and methods of the Jackson Heart Study Ethn Dis 2005 15 4 Suppl 6 6 17 16320381

[B33] Dubbert PM Carithers T Ainsworth BE Taylor HA Jr Wilson G Wyatt SB Physical activity assessment methods in the Jackson Heart Study Ethn Dis 2005 15 4 Suppl 6 6 16 16317986

[B34] Fuqua SR Wyatt SB Andrew ME Sarpong DF Henderson FR Cunningham MF Taylor HA Jr Recruiting African-American research participation in the Jackson Heart Study: methods, response rates, and sample description Ethn Dis 2005 15 4 Suppl 6 6 29 16317982

[B35] Carithers T Dubbert PM Crook E Davy B Wyatt SB Bogle ML Taylor HA Jr Tucker KL Dietary assessment in African Americans: methods used in the Jackson Heart Study Ethn Dis 2005 15 4 Suppl 6 6 55 16317985

[B36] Malina RM Tracking of physical activity and physical fitness across the lifespan Res Q Exerc Sport 1996 67 Suppl 3 48 S57 10.1080/02701367.1996.10608853 8902908

[B37] Tudor-Locke C Giles-Corti B Knuiman M McCormack G Tracking of pedometer-determined physical activity in adults who relocate: results from RESIDE Int J Behav Nutr Phys Act 2008 5 39 10.1186/1479-5868-5-39 18687137 PMC2527334

[B38] Tudor-Locke C Johnson WD Katzmarzyk PT Accelerometer-determined steps per day in US adults Med Sci Sports Exerc 2009 41 1384 1391 10.1249/MSS.0b013e318199885c 19516163

[B39] Tudor-Locke C Hatano Y Pangrazi RP Kang M Revisiting "how many steps are enough?" Med Sci Sports Exerc 2008 40 Suppl 7 537 S543 10.1249/MSS.0b013e31817c7133 18562971

[B40] Tudor-Locke C Leonardi C Johnson WD Katzmarzyk PT Church TS Accelerometer steps/day translation of moderate-to-vigorous activity Prev Med 2011 53 682 692 10.1016/j.ypmed.2011.01.014 21295063

